# Early virological failure and HIV drug resistance in Ugandan adults co-infected with tuberculosis

**DOI:** 10.1186/s12981-016-0128-5

**Published:** 2017-01-05

**Authors:** Amrei von Braun, Christine Sekaggya-Wiltshire, Alexandra U. Scherrer, Brian Magambo, Andrew Kambugu, Jan Fehr, Barbara Castelnuovo

**Affiliations:** 1 Infectious Diseases Institute, College of Health Sciences, Makerere University, Kampala, Uganda; 2 Division of Infectious Diseases and Hospital Epidemiology, University Hospital of Zurich, University of Zurich, Rämistrasse 100, 8091 Zurich, Switzerland; 3 Uganda Research Unit on AIDS, Medical Research Council, Ugandan Virus Research Institute, Entebbe, Uganda

**Keywords:** Tuberculosis/HIV co-infection, Treatment monitoring, Virological failure, HIV drug resistance, Efavirenz

## Abstract

**Purpose:**

This cross-sectional study took place in the integrated tuberculosis (TB) clinic of a large outpatient clinic for HIV-infected patients in Kampala, Uganda. The purpose of this study was to describe the proportion of TB/HIV co-infected adults with virological failure, type and frequency of HIV drug resistance-associated mutations, and the proportion of patients with suboptimal efavirenz levels.

**Methods:**

HIV-1 plasma viral loads, CD4 cell count measurements, and efavirenz serum concentrations were done in TB/HIV co-infected adults. Genotypic resistance testing was performed in case of confirmed virological failure.

**Results:**

After a median time on ART of 6 months, virological failure was found in 22/152 patients (14.5%). Of 147 participants with available efavirenz serum concentration, 26 (17.6%) had at least one value below the reference range, including 20/21 (95.2%) patients with confirmed virological failure. Genotypic resistance testing was available for 16/22 (72.7%) patients, of which 15 (93.8%) had at least one major mutation, most commonly M184V (81.2%) and K103NS (68.8%).

**Conclusion:**

We found a high proportion of TB/HIV co-infected patients with virological failure, the majority of which had developed relevant resistance-mutations after a median time on anti-retroviral treatment (ART) of 6 months. Virological monitoring should be prioritized in TB/HIV co-infected patients in resource-limited settings.

## Background

In 2013, the World Health Organization (WHO) reported 47′650 new cases of tuberculosis (TB) in Uganda, making it to one of 22 high-burden TB countries [1]. Of all new cases reported, 48% of the individuals were co-infected with Human Immunodeficiency Virus (HIV). Despite considerable improvements in care for both infections, such as enhanced access and earlier initiation of antiretroviral treatment (ART), as well as intensified TB case finding strategies, TB remains the leading cause of death among HIV-infected individuals in sub-Saharan Africa [2]. The main challenges in management of co-infection with *Mycobacteria tuberculosis* and HIV could be attributed to overlapping toxicities and drug–drug interaction [3], immune reconstitution inflammatory syndrome [4], costly treatment monitoring, and difficulties in adherence [5].

In resource-limited settings such as Uganda, close treatment monitoring strategies as optimally required for both infections are not readily available for all patients. For financial reasons, virological monitoring of HIV-infected patients on ART has either not been available or is reserved for selected patients (targeted viral loads), such as patients with diagnosed immunological failure [6]. While CD4 cell counts are available to patients free of charge, at the time of this study the facility price per viral load was approximately 60USD in Kampala.

In the absence of routine or targeted viral load testing, the WHO recommends to monitor ART using CD4 count testing [7], although immunologic criteria for treatment failure have a low sensitivity and positive predictive value for the detection of patients with virological failure [8]. Especially when TB co-infection is present, the interpretation of clinical and immunological findings while monitoring HIV-infected patients is challenging [9–11]. Individual patient HIV drug resistance testing is not routinely available due to its high costs.

Evidence of reduced morbidity and mortality supports early initiation of ART in TB co-infected patients [12]. According to WHO treatment recommendations [7], TB/HIV co-infected patients are preferably initiated on an efavirenz-based regimen within the first 8 weeks of TB treatment, due less drug–drug interactions when co-administered with rifampicin-based TB treatment and well-demonstrated efficacy [1].

So far, little information is available on early virological outcomes of TB/HIV co-infected patients on ART, and the development of HIV drug resistance in those with viral failure. The objective of this study was to assess the proportion of TB/HIV co-infected Ugandan patients with virological failure, identify factors potentially associated with treatment failure, and to report type and frequency of HIV drug resistance mutations detected in TB/HIV co-infected patients failing ART.

## Methods

This cross-sectional study was conducted at the integrated TB/HIV clinic of the Infectious Diseases Institute (IDI) in Kampala, Uganda, between April 2013 and 2015. The IDI currently provides care for over 8000 HIV-infected individuals. In the integrated TB clinic [13] approximately 300 cases of TB are diagnosed annually. At the time of this study, patients on ART were monitored with CD4 count measurements, although targeted viral load testing was available.

This study was a sub-study of the ongoing “Study on Outcomes related to tuberculosis and HIV drug concentrations in Uganda” (SOUTH study), which investigates the association between TB treatment outcome and serum concentrations of anti-TB drugs in TB/HIV co-infected Ugandan adults (ClinicalTrials.gov Identifier: NCT01782950). Participants included in the study were HIV-infected adults of 18 years and above, receiving treatment with rifampicin, isoniazid, pyrazinamide, ethambutol for 8 weeks, followed by 16 weeks of rifampicin/isoniazid for newly diagnosed pulmonary TB [14]. Efavirenz-based ART was started two to four weeks after initiation of TB treatment in previously ART-naïve patients. ART experienced patients on nevirapine-based ART at time of TB diagnosis were switched to efavirenz [14].

HIV type 1 plasma viral load measurements were performed as soon as a patient had been on ART for at least 6 months, accompanied by simultaneously measured CD4 cell count. Viral load measurements (COBAS® AmpliPrep/COBAS® TaqMan® HIV-1 Test, v2.0, Roche Diagnostics) and CD4 cell counts (BD FACSCalibur Flow Cytometer: 4-Color) were performed at the Makerere-University-John-Hopkins-University (MUJHU) CORE laboratory certified by the College of American Pathologists, located at the IDI. For patients receiving efavirenz-based ART, efavirenz serum concentrations were measured on-site, 9–15 h post-dosing using a validated ultra-violet high-performance liquid chromatography (UV-HPLC) method at week 8 and 24 of TB treatment. Patients with confirmed virological failure received genotypic drug resistance testing performed at the Ugandan Virus Research Institute/Medical Research Council (UVRI/MRC) in Entebbe, Uganda, (Method: PCR gel electrophoresis and purification (QIAquick PCR purification kit), sequencing (Big dye terminator v3.1 cycle sequencing kit, Applied Biosystems), genetic analysis (ABI 3500 and ABI 3130 machines, Applied Biosystems), base-called sequences (Sequencher v5.3 and sequence alignments, BioEdit v7.2.5 and SeaView v4.0), quality assurance (Calibrated Population Resistance tool, Stanford and the Los Alamos National database for the HIV Sequence Quality Analysis), and assigning of drug resistance mutations (submission of sequences to Stanford HIVdb Program).

Virological failure was defined as one HIV-1 plasma viral load >1000 copies/ml or two consecutive viral loads >400 copies/ml within three months. Favorable outcome of TB treatment was defined as fulfilling the WHO criteria of either cured or completed treatment [14]. History of HIV-infection, past and current ART regimens, and previous CD4 cell counts were derived from the routine electronic medical records and study case report forms. Characteristics of patients with virological treatment failure were compared to patients without treatment failure. Proportions were compared using Fisher’s exact test and medians using Wilcoxon rank-sum test.

## Results

During the study period, one hundred and fifty-two individuals were enrolled in this study. The median age was 34 years (interquartile range (IQR): 28–39) and 58.6% of the study participants were male. The majority was ART-naïve at time of TB diagnosis (110, 72.4%) and they were initiated on efavirenz-based ART two to four weeks after starting TB treatment. The median CD4 cell count at time of viral load measurement was 285 cells/µl (IQR: 150–453).

The most common ART regimen at time of viral load measurement consisted of tenofovir, lamivudine and efavirenz (119 patients, 78.3%), followed by zidovudine, lamivudine and efavirenz (29 patients, 19.1%). Only four patients (2.6%) were on second-line treatment with tenofovir, lamivudine and either ritonavir-boosted atazanavir or ritonavir-boosted lopinavir. Of all patients enrolled, 30 (19.7%) had previously substituted at least one antiretroviral drug, mainly due to TB co-infection.

Viral load measurements were performed after a median time on ART of 6 months (IQR: 5.5–12). Virological failure was found in 22/152 (14.5%) patients, with a median viral load log_10_ of 4.6 copies/ml (IQR: 4.0–5.3). Of patients with virological failure, 11 (50%) had been ART-naïve at TB diagnosis. Table 1 compares characteristics of patients with and without virological failure. There was no difference in ART regimens between the two groups, however, a higher proportion of patients with virological failure had one or more antiretroviral drug substitution in the past as compared to patients with no virologic failure (history of ART substitution: 36.4 vs. 17.0%, p value: 0.044).

**Table 1:** Characteristics of tuberculosis (TB)/HIV co-infected patients stratified by virological outcome with and without virological failure

Characteristics	All patients n = 152	Viral suppression n = 130	Virological failure n = 22	P value
Male gender [N, %]	89 (58.6)	80 (61.5)	9 (40.9)	0.099
Age (years) [Median, IQR]	34.1 (27.0–39.4)	34 (28.3–38.9)	34.1 (25.2–40.9)	0.882
CD4 cell count (cells/µl) [Median, IQR]	285 (150–453)	317 (170–465)	130 (43–341)	<0.001
Time on ART (months) [Median, IQR]	6 (5.5–12)	6 (5.5–11)	6 (5–24)	0.157
ART regimen [N, %]				0.194
TDF-3TC-EFV	119 (78.3)	105 (80.8)	14 (63.6)	
AZT-3TC-EFV	29 (19.1)	22 (16.9)	7 (31.8)	
TDF-3TC-ATV/r	3 (1.9)	2 (1.5)	1 (4.6)	
TDF-3TC-LPV/r	1 (0.7)	1 (0.8)	0	
History of ART switch [N, %]	30 (19.7)	22 (17)	8 (36.4)	0.044
Immunological failure [N, %]	21 (13.8)	10 (7.7)	11 (50)	<0.001
EFV serum concentration <1 mg/l [N/, %]	26 (17.7)	6 (4.8)	20 (95.2)	<0.001
TB outcome [N, %]				0.004
Cured/completed	142 (93.4)	125 (96.2)	17 (77.3)	
Failure	3 (2.0)	1 (0.8)	2 (9.1)	
Died	2 (1.3)	2 (1.5)	0	
Unknown	5 (3.3)	2 (1.5)	3 (13.6)	

*TDF* tenofovir, *3TC* lamivudine, *EFV* efavirenz, *AZT* zidovudine, *ATV/r* ritonavir-boosted atazanavir, *LPV/r* ritonavir-boosted lopinavir, *Immunological failure* drop of CD4 cell count to or below baseline, *Virological failure* HIV plasma viral load >1000 copies/ml or two consecutive viral loads >400/ml within 3 months

Generally, TB treatment outcome was favorable in our study population with 142 (93.4%) patients being cured or completed treatment. However, a lower proportion of patients with virological failure had a favorable TB treatment outcome (77.3%) as compared to patients with viral suppression (96.2%) (p value: 0.004).

A total of 148 participants were on efavirenz-based ART, of which 147 had available efavirenz serum concentration. Of these, 26 (17.7%) had at least one efavirenz serum concentration below the reference range of 1 mg/l; a significantly higher proportion of patients with confirmed virological failure (95.2%) had a serum concentration below the reference range as compared to patients with viral suppression.

Of the 22 patients with virological failure, 16 (72.7%) underwent genotypic resistance testing. The remaining 6 patients were either lost-to-follow-up (2 patients), had died (2 patients), or were switched to second-line treatment before resistance testing could be done (2 patients). HIV subtype distribution of these 16 patients was as follows: subtype A, C, D and recombinant were 3, 1, 3, and 8 patients respectively. Among recombinant HIV strains we found 5 CRF and 3 A/D. As shown in Table 2, genotypic resistance testing revealed that 15 of 16 (93.8%) patients had developed one or more relevant resistance mutations to first-line drugs commonly used in Uganda. The most common mutations found were M184V (81.2%) reflecting treatment with the nucleoside/nucleotide reverse transcriptase inhibitor (NRTI) lamivudine, and K103NS (68.8%) reflecting prior treatment with the non-nucleoside reverse transcriptase inhibitors (NNRTI) nevirapine or efavirenz. Furthermore, 13/16 (81.3%) patients had developed two drug class resistance mutations to both NNRTI and NRTI, while only 2/16 (12.5%) had developed resistance mutations to only one drug class (NNRTI). Major resistance mutations to protease inhibitors were not observed in our study population. One patient on second-line treatment underwent resistance testing which revealed resistance to NNRTI only.

**Table 2:** Type and frequency of resistance-associated mutations detected in 16 patients with virological failure

Mutations	N (%)
Any major mutation	15 (93.8)
Any NRTI mutation	13 (81.2)
Any TAM	5 (31.3)
3 or more TAMs	3 (18.8)
M41L	2 (12.5)
K65R	4 (25.0)
D67N	2 (12.5)
K70R	2 (12.5)
K70E	1 (6.3)
L74V/I	1 (6.3)
Y115F	1 (6.3)
M184IV	13 (81.2)
L210W	1 (6.3)
T215FY	2 (12.5)
K219GE	4 (25)
NNRTI major mutations	
Any major mutation	15 (93.8)
L100I	0
K101PEH	3 (18.8)
K103NS	11 (68.8)
V106AM	1 (6.3)
E138KAGQ	2 (12.5)
V179DEF	1 (6.3)
Y181CIV	1 (6.3)
Y188LCH	1 (6.3)
G190ASEQ	5 (31.3)
F227LC	0
M230L	4 (25)
PI mutations	
Minor	9 (56.3)
Major	0

*NRTI* nucleoside/nucleotide reverse transcriptase inhibitors, *TAM* thymidine analogue mutations, *NNRTI* non-nucleoside/nucleotide reverse transcriptase inhibitors, *PI* protease inhibitor

## Discussion

Among our population of Ugandan TB/HIV co-infected adults on ART and TB treatment we found a high proportion (14.5%) of patients with virological failure after a median time on ART of 6 months. A similarly high proportion of treatment failure was previously observed among TB/HIV co-infected patients from India [15, 16]. However, the proportion of virological failure among patients without TB at our clinic was much lower (7.95%) [17]. Furthermore, the majority of patients presented here had already developed two-class drug resistance which strongly limits their treatment options in a resource-limited setting such as Uganda. TB treatment outcome overall was favorable, but a significantly smaller proportion of patients with virological failure was either cured or completed TB treatment as compared to virologically suppressed patients.

The majority of patients with virological failure on efavirenz-based ART had at least one efavirenz serum concentration below reference range during concomitant TB treatment. Efavirenz levels below reference range are a risk factor for virological failure and the development of resistance-associated mutations [18]. Although pharmacokinetic aspects such as drug–drug interactions [3] as well as pharmacogenetic factors [19, 20] can reduce efavirenz concentrations, it seems that in our population poor adherence was a major contributing factor to low efavirenz levels considering the fact that a lower proportion of patients with virological failure had a favorable TB outcome compared to virological suppressed patients. This suggests this patient group may have been overall less compliant to both ART and TB treatment.

Reasons for poor adherence are diverse in TB/HIV co-infected patients and include large pill burden and significant side effects, as well as factors affecting non-TB-infected HIV-positive patients such as stigma or disbelief. As TB in sub-Saharan Africa disproportionately affects the economically most disadvantaged members of society [21], additional financial burdens due to lost wages through illness and frequent clinic visits as well as long distances to care centers may further challenge optimal adherence in this patient group.

Only 50% of patients with virological failure also had immunologic failure. Thus, in the absence of virological monitoring, treatment failure would not have been detected in these patients. Patients with undetected treatment failure are at risk of further accumulation of resistance mutations, which complicates future treatment in a setting with limited antiretroviral drug options and may contribute to the transmission of drug resistant viruses to partners. In our study population, the majority of patients with virological failure had already developed relevant resistance mutations after a median time on ART of 6 months. Among patients with available data on HIV drug resistance, 15/16 (93.8%) required switching to second-line drugs due to resistance-associated mutations. Most strikingly, the majority of the patients had developed two class drug resistance to both NNRTI and NRTI, which greatly limits optimal treatment choices in this setting. M184 V and K103NS were the most prevalent mutations found, reflecting prior treatment with lamivudine, efavirenz or nevirapine. Major PI mutations were not observed in any of the participants.

Partly, our study findings are limited by the fact that HIV drug resistance tests were not performed before the initiation ART and, therefore, information on transmitted drug resistance was not available. However, so far pre-treatment HIV drug resistance seems to be low in Uganda [22].

## Conclusion

In conclusion, based on our study finding of a high proportion of TB/HIV co-infected patients with virological failure and selection of relevant resistance mutations after only 6 months on ART, we recommend that virological monitoring should be prioritized in TB/HIV co-infected patients in resource-limited settings. In addition, further efforts are required to address specific adherence challenges in TB/HIV co-infected patients.

## Abbreviations

HIV: Human Immunodeficiency Virus
TB: tuberculosis
ART: anti-retroviral treatment
WHO: World Health Organization
IDI: Infectious Diseases Institute
SOUTH study: Study on Outcomes related to Tuberculosis and HIV drug concentrations in Uganda
MUJHU: Makerere-University-John-Hopkins-University
UV-HPLC: ultra-violet high-performance liquid chromatography
UVRI/MRC: Ugandan Virus Research Institute/Medical Research Council
IQR: interquartile range
NRTI: nucleoside reverse transcriptase inhibitors
NNRTI: non-nucleoside reverse transcriptase inhibitors
